# Effects of the Zbtb1 Gene on Chromatin Spatial Structure and Lymphatic Development: Combined Analysis of Hi-C, ATAC-Seq and RNA-Seq

**DOI:** 10.3389/fcell.2022.874525

**Published:** 2022-04-25

**Authors:** Junhong Wang, Chunwei Shi, Mingyang Cheng, Yiyuan Lu, Xiaoyu Zhang, Fengdi Li, Yu Sun, Xiaoxu Li, Xinyang Li, Yan Zeng, Chunfeng Wang, Xin Cao

**Affiliations:** ^1^ College of Veterinary Medicine, Jilin Agricultural University, Changchun, China; ^2^ Jilin Provincial Key Laboratory of Animal Microecology and Healthy Breeding, Jilin Agricultural University, Changchun, China; ^3^ Jilin Provincial Engineering Research Center of Animal Probiotics, Jilin Agricultural University, Changchun, China; ^4^ Key Laboratory of Animal Production and Product Quality Safety of Ministry of Education, Jilin Agricultural University, Changchun, China

**Keywords:** Zbtb1, EL4, Hi-C, ATAC-seq, RNA-seq

## Abstract

Zbtb1 (zinc finger and BTB domain containing 1) is a member of mammalian zbtb gene family. A series of bioinformatics analysis was carried out for the EL4 cell and the Zbtb1-deficient EL4 cell by Hi-C, ATAC-seq and RNA-seq techniques. Finally, Hi-C results showed that the intensity of chromatin interaction in the deletion group decreased with distance, the degree of chromosome interaction decreased significantly, the AB division region changed significantly, and the compactness of TAD structure decreased; The results of ATAC-seq showed that the open area and degree of chromatin in the deletion group decreased; 7778 differentially expressed mRNAs were found by RNA-seq. Our experimental results for the first time expounded the significance of Zbtb1 gene for T cell development, lymphocyte production and apoptosis from the aspects of chromosome spatial structure and chromatin opening degree, and provided relevant theoretical basis and data support for the in-depth study of related Zbtb1 genes in the future.

## Introduction

Zbtb1 plays a key role in T cell development and lymphocyte development, mRNA encoding Zbtb1 is most highly expressed in hematopoietic stem cells, thymocytes and pre-B cells, In addition to its role in T cell development, it was also demonstrated to be involved in the differentiation of B cells and NK cells, homozygous knockout of the Zbtb1 gene leads to severe combined immune deficiency in mice (Punwani et al., 2012; Lu et al., 2017). In other areas, acts as a transcriptional repressor (Matic et al., 2010); Represses cAMP-responsive element (CRE)-mediated transcriptional activation (Liu et al., 2011); Has a role in translesion DNA synthesis. Requires for UV-inducible RAD18 loading, PCNA monoubiquitination, POLH recruitment to replication factories and efficient translesion DNA synthesis (Kim et al., 2014). Our previous experimental results showed that Zbtb1 gene deletion slowed the growth rate of EL4 cells (Wang et al., 2021a).

Hi-C technology, derived from (Chromosome Conformation Capture—3C) technology, uses high-throughput sequencing technology, using proximity ligation combined with high-throughput sequencing, to study the interaction of the entire chromatin DNA on a genome-wide scale, taking the entire cell nucleus as the object of study (van Berkum et al., 2010). The formation of chromatin interactions is essential for the normal function of cells for the normal function of cells (Lafontaine et al., 2021). Hi-C data analysis is able to obtain information on interactions between genomic loci, divide the genome into bins of a specific size, and thus measure the strength of the interaction between two genomic loci (bins) (Fortin and Hansen, 2015).

ATAC-seq (Assay for Transposase-Accessible Chromatin with high-throughput sequencing) uses the preference for of open region chromatin to transposase for open region identification, and uses modified Tn5 transposase to directly introduce sequencing junctions into the open chromatin region by transposition reaction, and amplifies and sequences the open chromatin to finally obtain a genome-wide open chromatin map (Buenrostro et al., 2015).

Hi-C interaction data were analyzed jointly with ATAC-seq and transcriptome data, which can elucidate the mechanisms involved in organismal trait formation in terms of gene regulatory networks and epigenetic networks.

## Results

### Basic Quality-Related Data

Our sequencing analysis of Hi-C, ATAC-seq and RNA-seq was performed with the assistance of Annoroad Gene Technology (Beijing, China), and the related experimental methods are shown in (Supplementary Material S1), the preliminary quality statistics and extensive basic data data are shown in (Supplementary Material S2, S3, S4).

### Analysis of Hi-C Data Results

**FIGURE 1:**
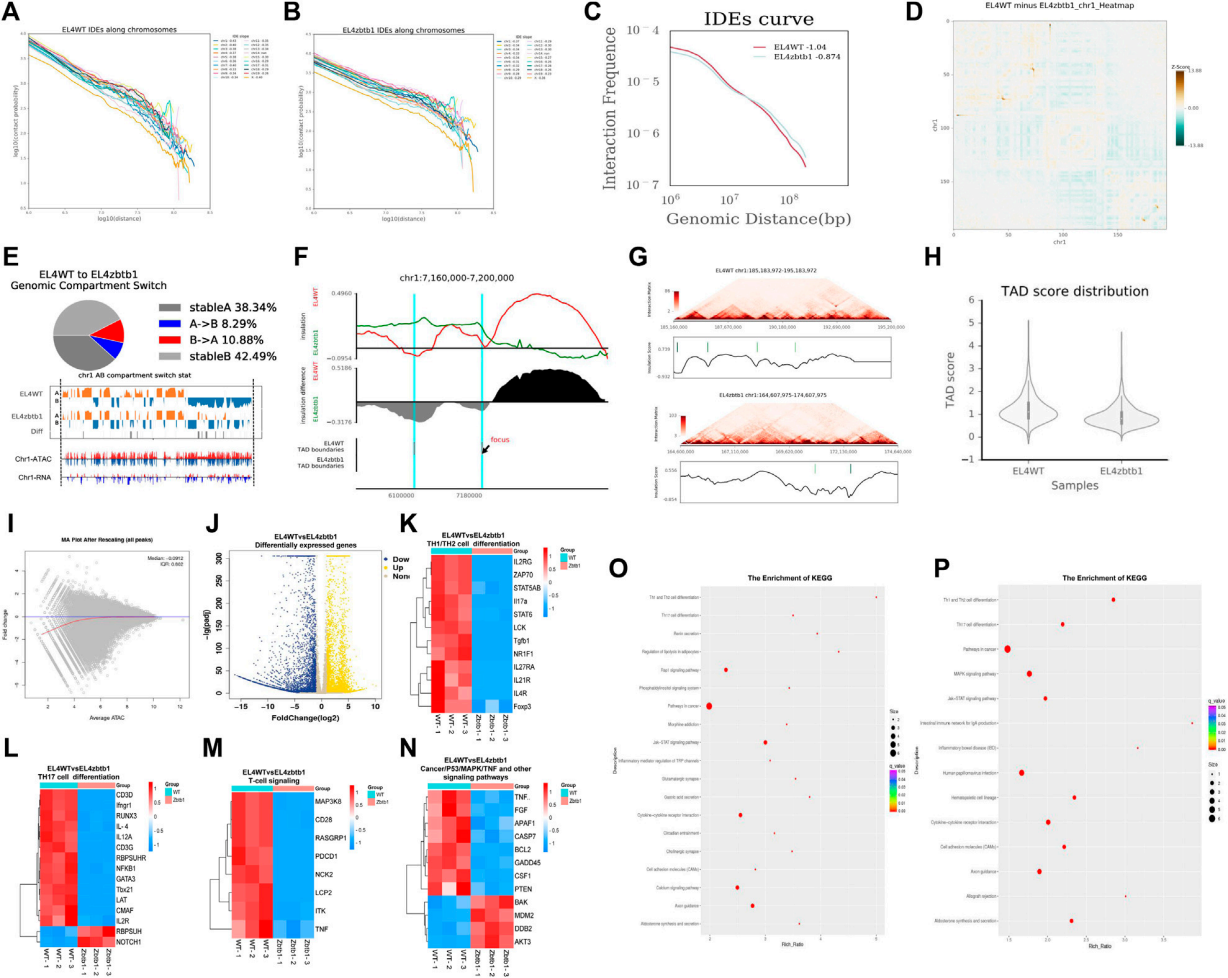
The analysis results of EL4 lacking zbtb1 were displayed by Hi-C, RNA-seq and ATAC-seq.

The results of the Hi-C experiment showed that the interaction ratio of CIS and trans chromosomes in the Zbtb1-deficient EL4 cell (KO group) and the EL4 cell (WT group) was approximately 75:25 (Supplementary Figure S1A). Chromatin interaction frequencies (IFS) attenuated with increasing distance in a given range, Decay of chromatin opening with distance in KO and WT groups (Figures 1A,B) and the interaction decay exponents (IDEs) of the KO group samples increased (Figure 1C). The interaction degree of chromosomes changed significantly, including CIS interaction changes as shown in Figure 1D (taking the first chromosome as an example) and trans interaction changes (shown in Supplementary Figure S1B). A/B components have cell specificity and can be transformed into different tissues and cells. This transformation is related to gene expression regulation (Chiliński et al., 2021). A comparison of the A/B component transformation between KO group and WT group is shown in Figure 1E, Also chromatin opening peaks and transcription of RNA were altered (taking the first chromosome as an example). In Hi-C interaction thermograms of mammals at around 40 kb resolution, we were able to observe that the thermograms show distinct triangular structures (Dixon et al., 2012). These triangular structures are named as Topologically associated domains (TAD), and these structural domains have distinct boundaries between them. The TAD structure is conserved in different times and spaces (organization, development stage, etc.), and there were also some dynamic changes. We found that the TAD structure of the cells without the Zbtb1 gene changed significantly, and the boundary was blurred or even disappeared, For example, the TAD change at position 7160000–7200000 on the first chromosome (Figure 1F). TAD display on the first chromosome of the WT and KO groups in Figure 1G. The TAD score, an index used to measure the tightness of the TAD structure, is shown in Figure 1H (Wang et al., 2021b).

### Analysis of ATAC-Seq Data Results

ATAC-seq results showed that the open peak regions in the deletion group cells were seriously reduced, and the openness of many regions was weakened. The chromatin open region map at the chromosome level in the whole genome is shown in Figure 1I visually shows the difference in the openness of all peaks, which is consistent with our RNA-seq verification results. There was a certain correlation between the differentially open region and the expression of differentially expressed mRNAs, the correlation was especially consistent between the open weakened region and the differentially downregulated mRNAs (Supplementary Figure S1C). The binding sites of transcription factors and other DNA sequences have certain characteristics, which are called Motifs, and therefore the detection of these Motifs in open regions of the whole genome can help to discover new transcription factors and annotate new functions of known transcription factors. We found that the Motifs of the KO group differed significantly from those of the WT group (Supplementary Material S5).

### Analysis of RNA-Seq Data Results

The RNA-seq results showed that 3185 differentially expressed mRNAs were upregulated and 4593 were downregulated. The volcanic map of the differentially expressed genes is shown in Figure 1J. We clustered the differentially expressed mRNA and found significant differences in the expression of key genes in Th1 and Th2 cell differentiation, Th17 cell differentiation and other pathways (Figures 1K–N). KEGG enrichment of genes annotated in chromosome differentially open regions and differentially expressed mRNAs (Figures 1O,P) found they were significantly enriched in lymphatic development-related pathways such as Th1 and Th2 cell differentiation, Th17 cell differentiation, etc. The signaling pathways related to cell growth, apoptosis and damage repair, such as MAPK, cancer pathways and TNF, were significantly enriched. According to the GO enrichment results (Supplementary Figure S1D), the differentially expressed mRNAs were enriched in 58 GO terms and significantly enriched in organelles, cell parts, cellular processes, biological regulation and binding.

### Combined Analysis of Hi-C, ATAC-Seq and RNA-Seq

**FIGURE 2:**
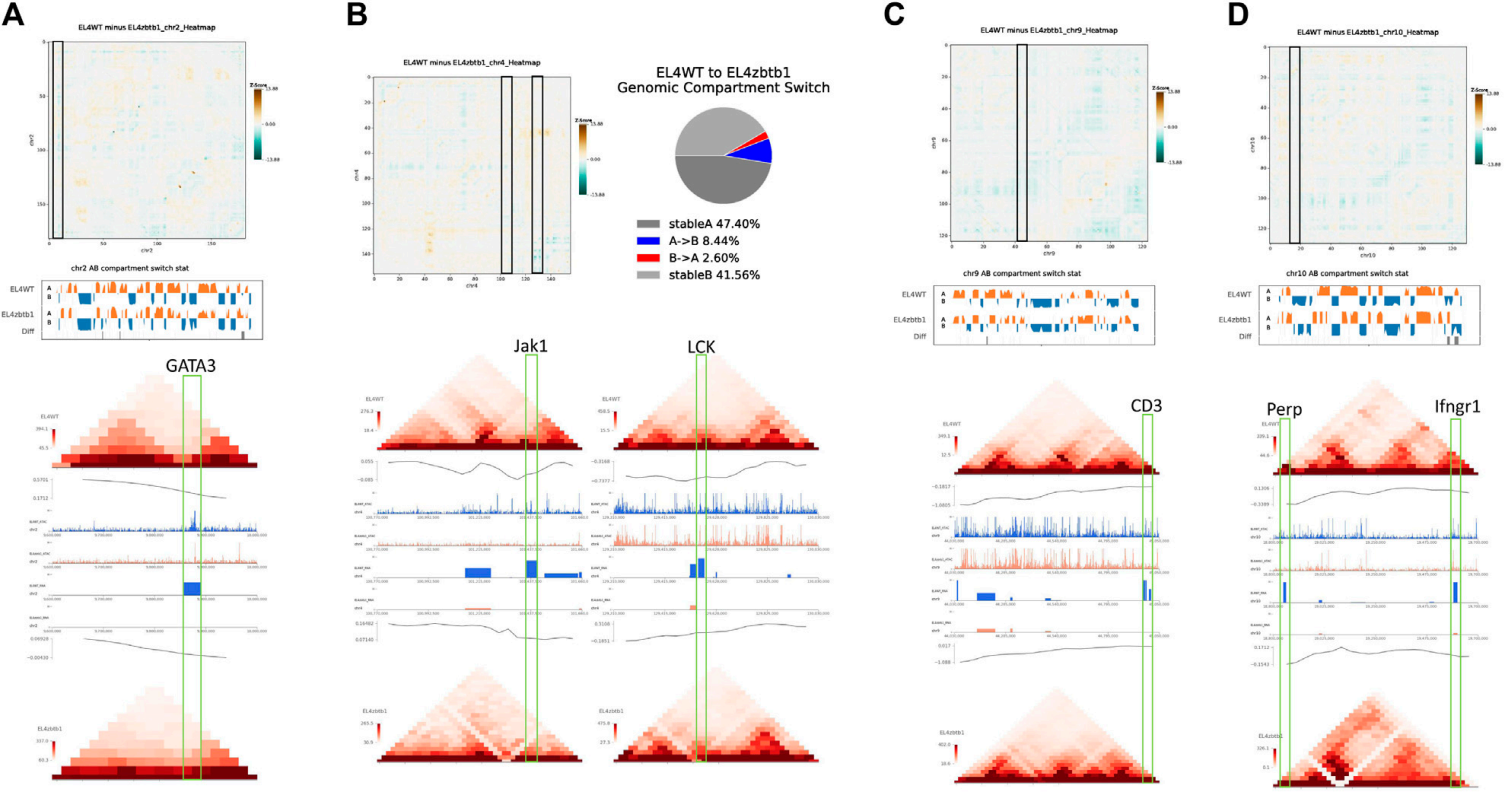
For the deletion of Zbtb1 gene the results of the association analysis demonstrated.

Previous studies have shown that Zbtb1 is a key determinant of T cell development and lymphocyte production and it affects cell apoptosis (Cao et al., 2016; Cheng et al., 2021). Through the combined analysis of Hi-C, ATAC-seq and RNA-seq, we found that KO group lost the expression of multiple key genes involved in Th1, Th2 and Th17 cell differentiation, T cell signaling and p53 signaling. Combined with the results of TAD, AB compartment and chromatin openness in the region where the genes are located, it was found that the degree of chromosome interaction at the location of the genes not being transcribed was generally reduced, the TAD boundary of the gene location became blurred, the open chromatin region was reduced, and the openness was weakened. Therefore, some mRNA cannot be transcribed normally and the transcriptional restriction of these genes might be the reason why mice with deletion of Zbtb1 gene cannot survive normally. The genes that were not being transcribed included regulatory genes important for lymphocyte development and differentiation, including GATA3 (Jiang et al., 2021) (Figure 2A), PDCD1 (Supplementary Figure S1E), RASGRP1 (Supplementary Figure S1F) and others, such as signal transduction-related genes lat (Supplementary Figure S1G), JAK1 and LCK (Figure 2B); cell receptor-related genes, such as CD3 (including CD3d, CD3g, CD3e and other genes) (Figure 2C), IL7R (Bevington et al., 2020) (Supplementary Figure S1H), IL2RG (Supplementary Figure S1I), and CD69 (Supplementary Figure S1J); regulatory factor genes, such as IL17a, IL17f (Supplementary Figure S1K) and RUNX3 (Supplementary Figure S1L); and isopathways of apoptosis, such as BCL2 (Supplementary Figure S1M), LCP2 (Supplementary Figure S1N), the target gene PERP of p53/p63 and the antitumor key gene IFNGR1 (Roberts and Paraoan, 2020) (Figure 2D).

## Discussion

Our results showed that the deletion of the Zbtb1 gene in EL4 cells led to the downregulation of the expression of many genes and great changes in the spatial structure of the chromatin. As a transcriptional repressor gene, Zbtb1 deletion also led to the upregulation of some genes. However, the ATAC results showed that the open chromatin region of many upregulated genes did not change significantly, which may be due to the restriction of gene expression due to transcriptional inhibition. After the deletion of the Zbtb1 gene, the inhibition was relieved, resulting in the upregulation of related gene expression, but it is difficult to say whether it is direct or indirect regulation.

Our experimental results for the first time explained the important effects of the Zbtb1 gene on T cell development, lymphocyte production and apoptosis from the aspects of chromosome structure and chromatin spatial changes, which provided a relevant theoretical basis and data support for a future in-depth study of Zbtb1.

## Data Availability

The datasets presented in this study can be found in online repositories. The names of the repository/repositories and accession number(s) can be found in the article/Supplementary Material.
