# Supplementary figures and images for: Effects of the Zbtb1 Gene on Chromatin Spatial Structure and Lymphatic Development: Combined Analysis of Hi-C, ATAC-Seq and RNA-Seq
Source: Front Cell Dev Biol. 2022 Apr 25;10:874525. doi: 10.3389/fcell.2022.874525 (PMC9081333; doi:10.3389/fcell.2022.874525)

A

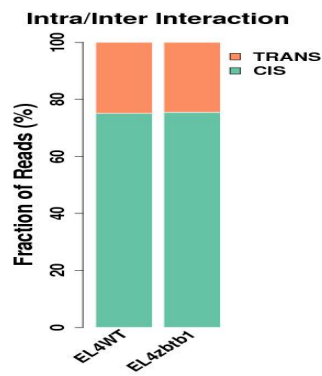

C

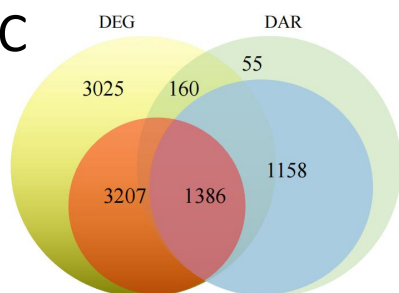

E

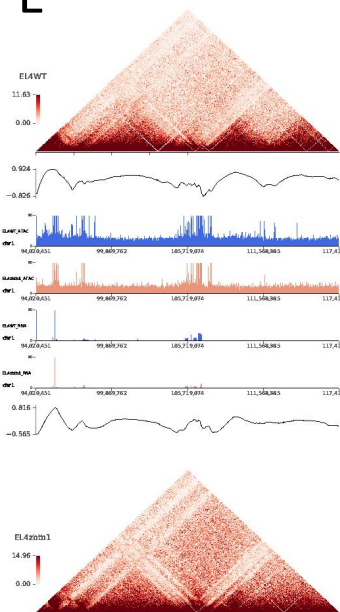

J

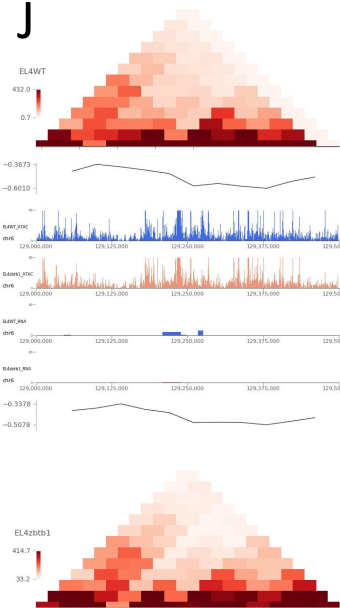

B

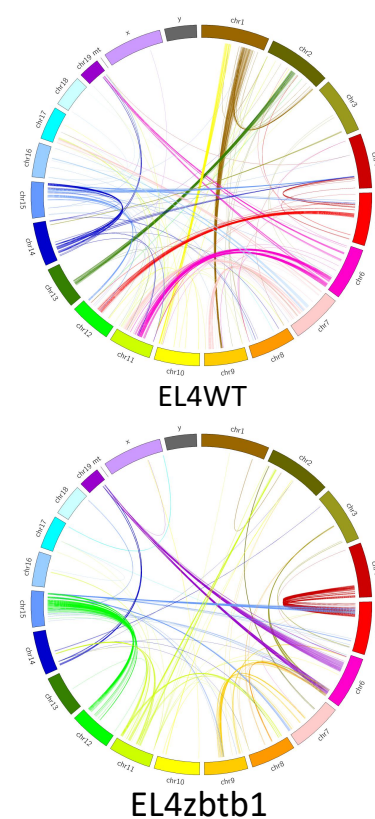

F

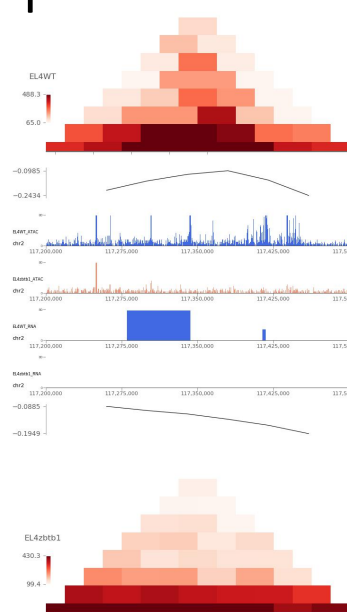

K

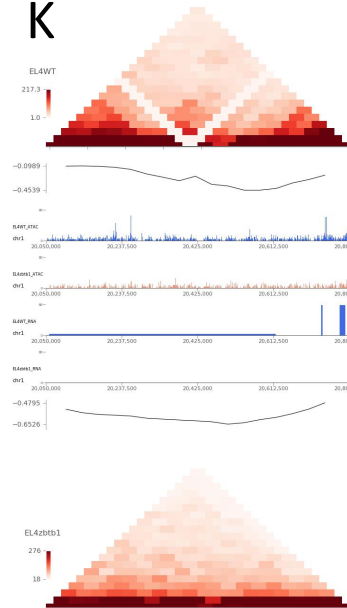

D

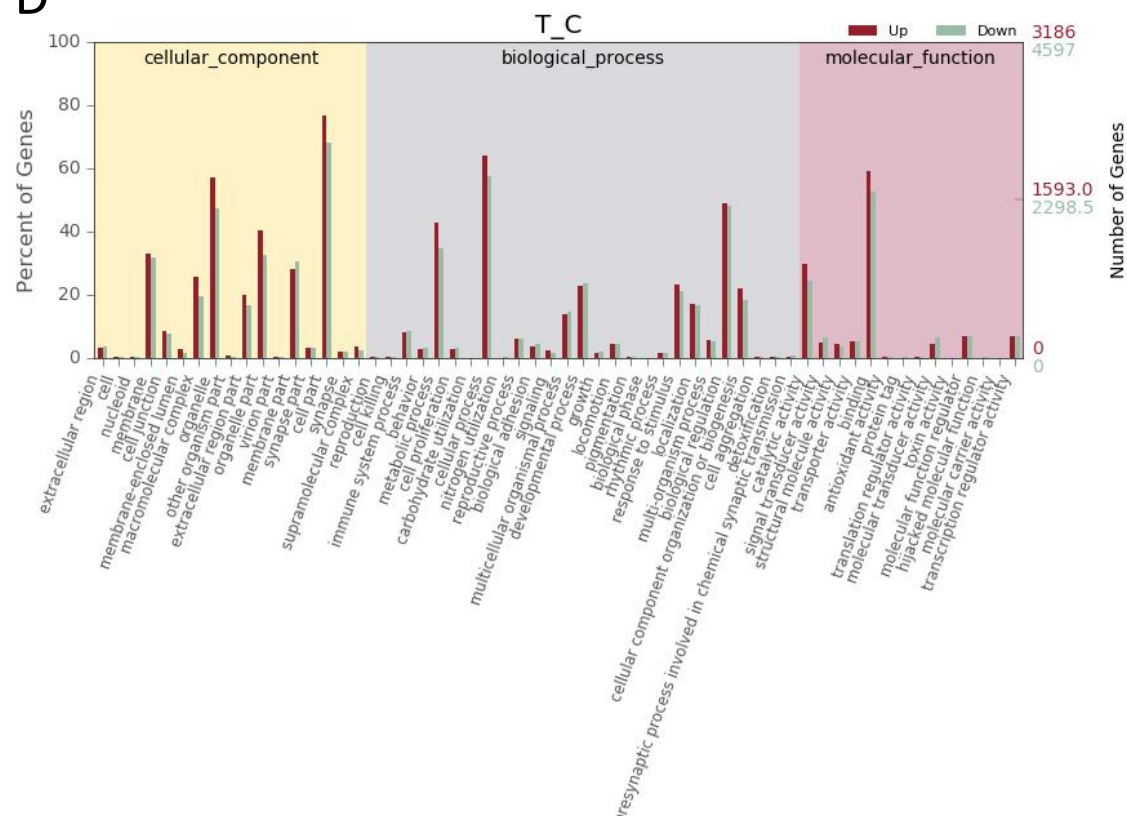

G

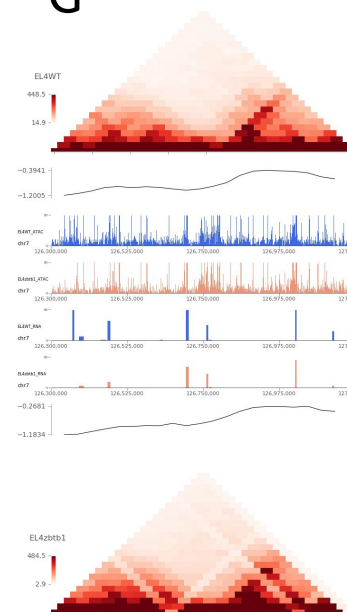

L

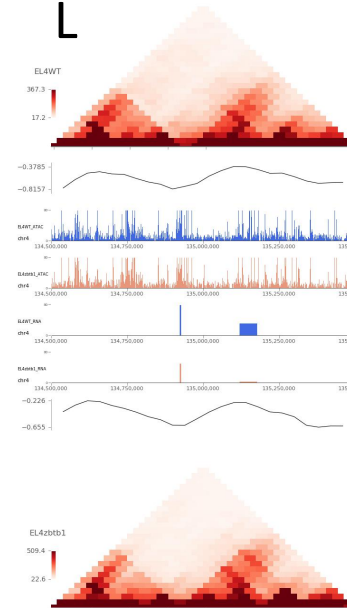

H

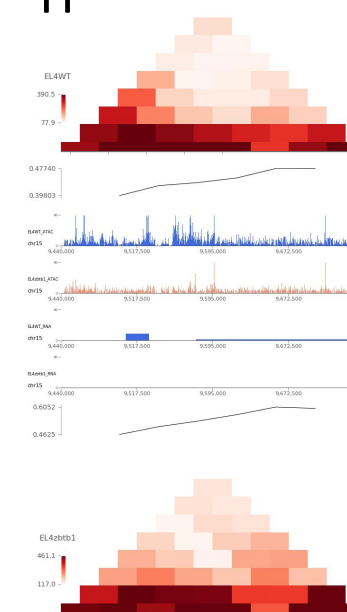

M

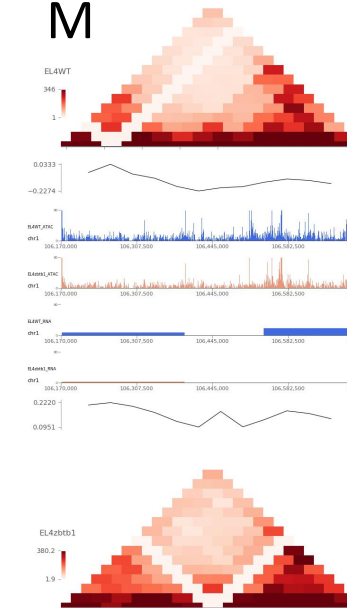

I

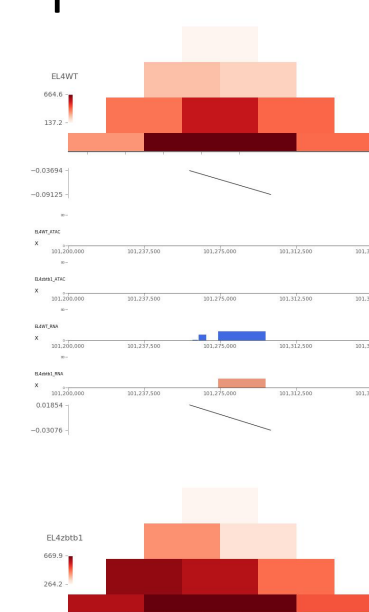

N

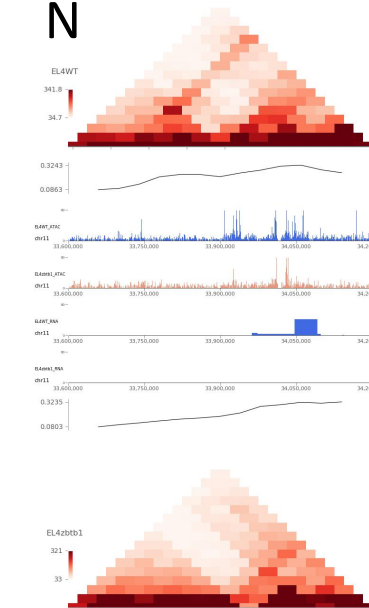

Supplement: Supplementary file 1 [file DataSheet1.PDF]
