# Supplementary material for: Effects of the Zbtb1 Gene on Chromatin Spatial Structure and Lymphatic Development: Combined Analysis of Hi-C, ATAC-Seq and RNA-Seq
Source: Front Cell Dev Biol. 2022 Apr 25;10:874525. doi: 10.3389/fcell.2022.874525 (PMC9081333; doi:10.3389/fcell.2022.874525)
Supplement: Supplementary file 2 [file DataSheet2.docx]

**Recommended sample collection, treatment, and shipment procedures for**

**standard Hi-C library preparation**

**1. Crosslinking of cells**

Grow the cells in appropriate culture medium. In general, 2.5 x 107 cells and the amount of genomic DNA more than 15 ug are enough for making one Hi-C library. However we suggest growing 5 x 107 to 1 x 108 cells for crosslinking to provide technical replicates if necessary. It is very important to estimate the number of cells accurately because it affects the complexity of the Hi-C library.

**For adherent cells:**

1.1) Aspirate the medium and add 22.5 ml of fresh medium without serum per plate.

1.2) Crosslink the cells by adding 1.25 ml of 37% formaldehyde to obtain 2% final

concentration. Mix gently, immediately after addition of formaldehyde.

1.3) Incubate at room temperature (RT) for exactly 10 min. Gently rock the plates

every 2 min.

1.4) To quench the crosslinking reaction, add 2.5 ml of 2.5 M glycine, mix well.

1.5) Incubate for 5 min at RT and then incubate on ice for at least 15 min to stop

crosslinking completely.

1.6) Scrape the cells from the plates with a cell scraper and transfer to a tube.

1.7) Mix cells well and then split the crosslinked cell suspension into aliquots of 25

x 106 cells.1.8) Centrifuge the crosslinked cells at 800xg for 10 min.

1.9) Discard the supernatant by aspiration. It is important to remove the liquid

phase completely.

1.10) Cells can be snap-frozen in liquid nitrogen and stored at -80°C for at least 1.5

years or one can continue with cell lysis.

**For suspension cells:**

1.1) Gently pellet the cells by spinning at 300xg for 10 min at RT.

1.2) Discard the supernatant.

1.3) Thoroughly resuspend the pellet in 42.5 ml of fresh culture medium without

serum. Break cell clumps by pipetting up and down.

1.4) Crosslink the cells by adding 2.5 ml of 37% formaldehyde in one shot (2% final

concentration). Mix quickly by inverting the tube several times.

1.5) Incubate at room temperature (RT) for exactly 10 min. Gently invert the tube

every 1-2 min.

1.6) To quench the formaldehyde in the reaction, add 5 ml of 2.5 M glycine in one

shot, mix well.

1.7) Incubate for 5 min at RT and then incubate on ice for at least 15 min to stop

crosslinking completely.

1.8) Split the crosslinked cell suspension into aliquots of 25 x 106 cells.

1.9) Centrifuge the crosslinked cells at 800xg for 10 min.

1.10) Discard the supernatant by aspiration. It is important to remove the liquid phase completely.

1.11) Cells can be snap-frozen in liquid nitrogen and stored at -80°C.

**2. storage and shipment**

Dry ice shipping is required for crosslinked cells.

**3. Hi-C library preparation**

The fixed cells were resuspended in 1 ml of lysis buffer (10 Mm Tris-HCl pH 8.0, 10 mM NaCl, 0.2% Igepal CA-630, 1/10 vol. of proteinase inhibitor cocktail (Sigma)), and then incubated on ice for 20 minutes. Nuclei were pelleted by centrifugation at 4 °C, 600x g for 5 minutes, and then washed with 1 ml of the lysis buffer, followed by another centrifugation under similar conditions. After washing twice with restriction enzyme buffer, the nuclei were resuspended in 400 μl of restriction enzyme buffer and transferred to a safe-lock tube. Next, The chromatin is solubilized with dilute SDS and incubation at 65 ℃ for 10 min. After Quenching the SDS by Triton X-100 Overnight digestion was applied with 4 cutter restriction enzyme (400 units MboI) at 37℃ on rocking platform. The next steps are Hi-C specific, including marking the DNA ends with biotin-14-dCTP and performing blunt-end ligation of crosslinked fragments. The proximal chromatin DNA was religated by ligation enzyme. The nuclear complexes were reversed crosslinked by incubating with proteinase K at 65℃. DNA was purified by phenol-chloroform extraction. Biotin-C was removed from non-ligated fragment ends using T4 DNA polymerase. Fragments was sheared to a size of 200-600 basepairs by sonication. The fragment ends were repaired by the mixture of T4 DNA polymerase,T4 polynucleotide kinase and Klenow DNA polymerase. Biotin labeled HiC sample were specifically enriched using streptavidin C1 magnetic beads. The fragment ends were adding A-tailing by Klenow(exo-) and then adding Illumina paired-end sequencing adapter by ligation mix. At last, the Hi-C libraries were amplified by 12-14 cycles PCR , and sequenced in Illumina HiSeq platform. Sequencing interacting partern was obtained by Illumina HiSeq instrument with 2×150-bp reads.

**Methods of ATAC-seq**

**Introduction to ATAC-aeq library building and sequencing process**

1. The number and activity of cells were detected by microscope, and 50000 cells were taken.

2. 1 × Lysis buffer was used to lyse cells and obtain nuclei. Truepreptm DNA library prep was used Kit V2 for Illumina completes the establishment of transposon library.

3. Qubit 3.0 fluorometer detects the mass concentration, and Agilent HS 2100 Bioanalyzer detects the mass concentration The molar concentration was detected by steponeplustm real time PCR system.

4. For the qualified library, pe150 was sequenced by Illumina hiseq x ten.


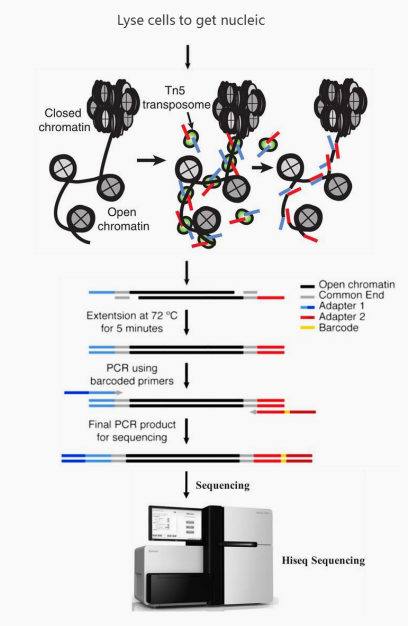


**Libraries preparation and sequencing**

Around 50,000 living cells were taken for each library preparation. The cells were lysed in 1 ×Lysis Buffer to get the nuclei, and TruePrep™ DNA Library Prep Kit V2 for Illumina (Vazyme Biotech) was used to construct the transposase-treated libraries. The mass concentration and molar concentration of libraries were detected by Qubit 3.0 Fluorometer and StepOnePlus™ Real-Time PCR system, respectively, and lengths of inserted fragments were detect with Agilent HS 2100 Bioanalyzer. Qualified libraries were sequencing by Illumina HiSeq X ten platform in pair-end 150 bp style.

**Bioinformation analysis**

Raw data was stored in FASTQ format, including the base sequence and corresponding quality information. Trimmomatic (v0.36) was used to remove adaptor-polluted or low-quality bases, and reads too short ( < 36 nt) were filtered out to get the clean data. Clean data was mapped to reference genome by Bowtie2, and visualized by IGV (Integrative Genomics Viewer). Peaks corresponding to the open region in genome were detected by MACS2, and significantly different peaks between samples were acquired using MAnorm. The enrichment analysis of GO term (http://geneontology.org/) or KEGG pathway (http://www.kegg.jp/) was based on hypergeometric test with the threshold q < 0.05, to find the significant enrichment of detected genes.

**Introduction to Experimental Methods for mRNA**

**sequencing**

**RNA quality examination**

RNA purity was checked using the kaiaoK5500®Spectrophotometer (Kaiao, Beijing, China).

RNA integrity and concentration was assessed using the RNA Nano 6000 Assay Kit of the Bioanalyzer 2100 system (Agilent Technologies, CA, USA).

**Library preparation for RNA sequencing**

A total amount of 2 μg RNA per sample was used as input material for the RNA sample preparations. Sequencing libraries were generated using NEBNext® Ultra™ RNA Library Prep Kit for Illumina® (#E7530L, NEB, USA) following the manufacturer’s recommendations and index codes were added to attribute sequences to each sample. Briefly, mRNA was purified from total RNA using poly-T oligo-attached magnetic beads. Fragmentation was carried out using divalent cations under elevated temperature in NEBNext First Strand Synthesis Reaction Buffer (5X). First strand cDNA was synthesized using random hexamer primer and RNase H. Second strand cDNA synthesis was subsequently performed using buffer, dNTPs, DNA polymerase I and RNase H. The library fragments were purified with QiaQuick PCR kits and elution with EB buffer, then terminal repair,A-tailing and adapter added were implemented. The aimed products were retrieved and PCR was performed, then the library was completed.

**Library examination**

RNA concentration of library was measured using Qubit® RNA Assay Kit in Qubit® 3.0 to preliminary quantify and then dilute to 1ng/μl. Insert size was assessed using the Agilent Bioanalyzer 2100 system (Agilent Technologies, CA, USA), and qualified insert size was accurate quantification using StepOnePlus™ Real-Time PCR System (Library valid concentration＞10 nM).

**Library clustering and sequencing**

The clustering of the index-coded samples was performed on a cBot cluster generation system using HiSeq PE Cluster Kit v4-cBot-HS (Illumina) according to the manufacturer’s instructions. After cluster generation, the libraries were sequenced on an Illumina platform and 150 bp paired-end reads were generated.
